# Exploring the Pivotal Functions of Tertiary Lymphoid Structures in Cancer Prognosis and Immunotherapy Outcomes

**DOI:** 10.3390/cancers17233754

**Published:** 2025-11-24

**Authors:** Constantin N. Baxevanis, Michael Sofopoulos, Ourania E. Tsitsilonis, Angelos D. Gritzapis

**Affiliations:** 1 Cancer Immunology and Immunotherapy Center, St. Savas Cancer Hospital, 171 Alexandras Avenue, 11522 Athens, Greece; 2 Flow Cytometry Unit, Department of Biology, National and Kapodistrian University of Athens, Panepistimiopolis, Ilissia, 15784 Athens, Greece; rtsitsil@biol.uoa.gr; 3 Department of Pathology, Andreas Syggros Hospital of Cutaneous and Venereal Diseases, Ionos Dragoumi 5, 16121 Athens, Greece; msofopoulos@med.uoa.gr

**Keywords:** tertiary lymphoid structures, tumor-infiltrating lymphocytes, antitumor immunity, chemokines, immune checkpoint inhibitors, immunotherapy

## Abstract

Tertiary lymphoid structures (TLS) arise from lymphoid neogenesis in non-lymphoid organs and are driven by persistent inflammation mediated by chemokines and cytokines. In cancer, TLS orchestrate immune mechanisms relevant to tumor growth and thereby influence clinical outcomes. Observations of a correlation between the presence of TLS and clinical benefits in cancer patients, which suggests that TLS may serve as prognostic and predictive biomarkers, have prompted increased investigation of TLS in tumors. Accumulating evidence indicates that the prognostic and predictive value of TLS is context-dependent and relevant to their cellular composition and functional state. Combining assessments of tumor-infiltrating lymphocytes (TILs) with TLS features yields a more refined prognostic tool by capturing both local immune activity and aspects of the broader tumor immune microenvironment. Improved understanding of TLS biology and of their interactions with TILs can enhance predictions of therapeutic response. Furthermore, therapeutic modulation of TLS composition or function to favor antitumor immunity represents a promising strategy to improve treatment outcomes.

## Simple Summary

Tertiary lymphoid structures (TLS) are immune cell hubs that form in non-lymphoid tissues during chronic inflammation. In tumors, they shape antitumor immune responses and are often linked with better patient outcomes, thus they are being studied as potential prognostic and predictive biomarkers. Their true value depends on their cellular makeup and activity, so combining TLS features with the levels of tumor-infiltrating lymphocytes gives a clearer picture of the tumor immune environment. Understanding and therapeutically shifting TLS toward an antitumor state could improve patients’ responses to treatment.

## 1. Introduction

The immune composition of the tumor microenvironment (TME) plays a central role in controlling tumor progression. Although tumor-draining lymph nodes have long been recognized as the primary sites for the generation of an effective antitumor adaptive immunity, recent studies have highlighted an important role for TLS in eliciting such responses [1,2]. TLS are ectopic lymphoid organs that arise under conditions of chronic inflammation, including autoimmune diseases and cancer [3,4]. Their cellular architecture typically includes high endothelial venules (HEVs), T and B lymphocytes, plasma cells, and dendritic cells (DCs) [5].

TLS can both promote and regulate antitumor immunity; their prognostic significance depends on density, maturity, and tumor type. Immature TLS that lack an organized structure or functional germinal centers (GCs) may be associated with weaker immune responses, whereas mature TLS, characterized by a GCs formation and active immune–cell interactions, are generally linked to improved clinical outcomes [5,6]. Stepwise TLS maturation requires the establishment of homeostatic chemokine gradients (e.g., CCL19, CCL21, CXCL13) that delineate compartmentalized zones for emerging lymphoid follicles [2,6]. As sites of antigen presentation and lymphocyte activation, TLS facilitate the generation of tumor-specific immune responses and often correlate with increased infiltration of cytotoxic CD8^+^ T cells into the TME, a key determinant of antitumor activity [6,7,8,9]. To date, TLS presence in the TME has most frequently been associated with favorable clinical outcomes across multiple malignancies [10,11,12,13,14]. We [15] and others [16,17,18] have reported the prognostic relevance of TLS in specific breast cancer (BCa) subtypes, particularly in triple-negative (TNBC) and HER2/neu-positive BCa, when considered together with TILs. Another BCa study has reported correlations between TLS and higher tumor grades [19]. We further demonstrated that integrating densities and the locations of tumor-infiltrating cytotoxic CD8^+^ T cells and suppressive CD163^+^ macrophages with TLS quantity and distribution improves prognostic accuracy in BCa, underscoring the importance of dynamic interactions among immune cells within the TME and TLS in determining clinical outcomes [20].

The abundance and maturation state of TLS may also predict patients’ responses to immunotherapy. Tumors rich in TLS are often more responsive to immune checkpoint inhibitors (ICIs) such as anti-programmed cell death protein 1 (PD-1) and anti-PD-1 ligand (PD-L1) therapies, likely because TLS prime and sustain T-cell-mediated immunity. For example, increased TLS numbers have been associated with improved clinical responses to neoadjuvant chemotherapy combined with immunotherapy in lung cancer [21]. In gastric cancer, TLS levels outperformed microsatellite instability and Epstein–Barr virus status in predicting benefits from anti-PD-1 therapy [22]. A TLS signature was upregulated after treatment with durvalumab plus oleclumab or durvalumab plus monalizumab in treatment-naïve non-small cell lung cancer (NSCLC) patients [23]. However, TLS are not universally prognostic of positive outcomes: in malignancies such as hepatocellular carcinoma and colorectal cancer, TLS may reflect chronic inflammation or the activation of immunosuppressive pathways rather than effective antitumor immunity [7,24]. In hepatocellular carcinoma, TLS within the liver parenchyma have been proposed to serve as microniches for tumor progenitor cells and to promote cancer cell stemness [7]. TLS can also harbor regulatory T cells (Tregs) or myeloid-derived suppressor cells (MDSCs), which dampen antitumor responses and may contribute to disease progression [6,20].

Overall, the prognostic impact of TLS depends on their maturity, cellular composition, and local signaling milieu. Chemokine gradients that recruit and organize DCs, B cells, and T follicular helper (Tfh) cells promote GCs-like activity and efficient antigen presentation, while abundant cytotoxic CD8^+^ T cells within or adjacent to TLS support effective tumor control. By contrast, Treg and MDSC enrichment of TLS can suppress antitumor responses and negate benefit. Finally, TLS location, density, and the relative balance between effector and suppressive TIL populations, factors that can be reshaped by therapy, collectively determine whether TLS predict favorable or poor outcomes. In this review, we examine pathways that govern TLS formation and function, assess their prognostic value both independently and in combination with TILs across human cancers, and consider therapeutic strategies to harness and augment TLS-driven antitumor immunity.

## 2. TLS Formation and Function

TLS form in response to persistent antigenic stimulation, a feature common to autoimmune and chronic inflammatory conditions. Structurally and functionally, TLS resemble secondary lymphoid organs, with distinct T- and B-cell zones, follicular dendritic cells (FDCs), fibroblastic reticular cells (FRCs), and lymphatic networks [5,6,7]. TLS are present in the TME of most malignancies, including BCa, colorectal cancer, gastric cancer, NSCLC, ovarian cancer, and melanoma, while their prevalence in renal cell carcinoma is relatively low [25,26,27,28]. High HEVs are specialized vascular structures within TLS that permit efficient recruitment of T and B cells [29,30,31]. Homeostatic chemokines such as CCL19, CCL21, CXCL13, and CXCL12, produced by FRCs and FDCs, are central in organizing the TLS microenvironment and maintaining its function [32,33,34]. CD4^+^ Tfh cells are essential TLS components because they drive GCs formation and support the differentiation of memory B cells and antibody-secreting plasma cells [1,9]. CXCL13-producing Tfh cells among TILs contribute substantially to immune-cell recruitment into the TME, promoting TLS development and maturation and thereby creating a niche that supports durable antitumor responses [35].

Although TLS are often associated with favorable clinical outcomes, individual TLS components can have opposing roles, so specific structural and functional features are required for TLS to be protective [6,36]. Tumors vary widely in the number, size, cellular composition, spatial distribution, and maturation state of their TLS, and each of these attributes can influence clinical impact independently [2,5]. For example, B cell clones within TLS can mature into plasma cells that produce IgG or IgA antibodies directed against tumor-associated antigens [1,9]. Mature TLS that contain abundant B cells and plasma cells producing tumor-specific antibodies are frequently linked to improved prognosis and better responses to immunotherapy [1,9,37,38,39,40]. Conversely, polyclonal IgG responses can form immune complexes that activate phagocytic macrophages to secrete inflammatory cytokines, which may promote angiogenesis and tumor progression [9,40,41]. In the absence of mature TLS, B cells are often sparse and ineffective or may differentiate into regulatory subsets with suppressive phenotypes [1,41]. A suppressive role for B-lineage cells has been observed in castration-resistant prostate cancer, whereby an abundance of plasma cells within TLS correlated with a worse prognosis; these B cells displayed immunosuppressive activity that was dependent on transforming growth factor-β receptor signaling and were positive for interleukin (IL)-10 and PD-L1 [42]. Increased angiogenesis can also remodel the TME by reducing TLS density and increasing the prevalence of immature DCs, M2-skewed macrophages, Tregs, and poorly cytotoxic CD8^+^ T cells, thereby creating a milieu that supports immune suppression and tumor growth. This phenotype has been reported across several cancer types and is often associated with PD-L1-expressing immune cells and tumor cells [43,44,45,46,47,48].

Taken together, these observations indicate that TLS organization and maturation critically determine whether TLS support durable antitumor immunity or, counterintuitively, contribute to immune suppression and tumor progression. Key structural elements, including HEVs, organized T- and B-cell zones, FDCs/FRCs, homeostatic chemokines, and CXCL13-producing Tfh cells, foster GCs-like reactions, tumor-specific antibody production, and improve clinical outcomes when present in a mature form. By contrast, immature or dysregulated TLS, angiogenesis-driven remodeling, or regulatory B cell phenotypes can undermine antitumor immunity and worsen prognosis. Because TLS attributes (number, size, composition, spatial localization, and maturation state) vary widely across tumor types and among patients, dissecting the features that define protective versus pathogenic TLS is essential. Translational efforts that preserve or induce mature, functionally competent TLS while limiting angiogenic and regulatory pathways that favor suppression, hold promise to improve responses to immunotherapy, but they will require precise biomarkers and mechanistic studies to guide therapeutic modulation.

## 3. The Prognostic Role of TLS

The prognostic significance of TLS stems from the idea that, like lymph nodes, they can promote antitumor responses within the TME. By shaping the immune composition of the TME, TLS create a permissive niche for immune-cell accumulation and the initiation of antitumor immunity. TLS promote CD8^+^ T-cell-mediated responses through multiple mechanisms, including the recruitment of CD4^+^ T cells, the secretion of chemokines, and the expression of adhesion molecules and integrins [49]. The involvement of mature DCs in these mechanisms is indispensable: we previously showed that mature DCs present tumor-derived peptides on major histocompatibility complex (MHC) class II molecules to stimulate CD4^+^ T cells, thereby facilitating subsequent CD8^+^ T-cell-mediated destruction of autologous tumor cells [50]. In NSCLC, high frequencies of TLS containing mature DCs define a TME biased toward antitumor immunity, characterized by the upregulation of genes involved in both CD4^+^ and CD8^+^ T cell activation and cytotoxic function, and associated with improved patient survival [51]. Consistent with this, several studies have linked dense, mature TLS to increased numbers of T cells expressing the transcription factor T-bet or to elevated cytotoxic gene signatures in NSCLC, colorectal cancer, and other malignancies [25,51,52]. Additional reports found that higher densities of T-bet^+^ TIL, together with mature DCs, correlate with favorable outcomes in gastric, colorectal, and cervical cancers [37,53,54]. These observations point to an important regulatory role for TLS-resident mature DCs in shaping the immune contexture of the TME.

Clinical data further support the prognostic role of TLS. Calderaro et al. [55] demonstrated that the presence of TLS in the TME reduced the risk of early relapse after surgery for hepatocellular carcinoma, suggesting active immune surveillance against residual tumor cells. Multiple studies have also linked HEVs and various TLS-resident immune populations, such as follicular B cells, Tfh cells, and mature DCs, to patient prognosis [25,36,56]. High TLS density is frequently associated with increased numbers of CD8^+^ effector-memory T cells and the upregulation of T-cell-activation pathways [35,51,53,57]. In colorectal cancer, TLS in primary tumors have been reported as a favorable prognostic marker, although stromal suppressive factors can mitigate this benefit [58,59]. Improved clinical outcomes after neoadjuvant chemotherapy in early-stage esophageal squamous cell carcinoma correlated with abundant mature TLS enriched in follicular DCs and Tfh cells and with increased densities of mature B cells [60]. Similarly, a phase II trial of neoadjuvant nivolumab in melanoma found that responders exhibited higher intratumoral densities of mature B cells and TLS and increased expression of B cell functional gene programs compared to non-responders [61].

In conclusion, the evidence supports a model in which TLS, particularly those enriched for mature DCs, act as intratumoral immune hubs that prime and sustain coordinated adaptive immune responses, thereby shaping a TME biased toward effective antitumor immunity and improving clinical outcome across multiple cancers. The consistent association between mature, densely organized TLS and a favorable prognosis, a response to chemotherapy and checkpoint blockade, and the enrichment of cytotoxic and T-bet-expressing lymphocytes underscores the potential of TLS density and composition as both prognostic biomarkers and therapeutic targets. Translating this promise will require mechanistic studies to define the drivers of TLS maturation, the standardization of TLS assessment in clinical specimens, and rational strategies to induce or harness mature TLS alongside existing immunotherapies, to achieve durable patient benefit.

## 4. The Combined Assessment of TILs and TLS as Indicators for Prognosis

Tumor-infiltrating cytotoxic CD8^+^ T cells are widely regarded as central effectors of intratumoral antitumor immunity and carry strong prognostic significance, often correlating with favorable clinical outcomes [62]. However, their functional programs are frequently impaired within the TME, suggesting that additional immune pathways and cell types contribute importantly to antitumor responses [63,64]. Combining TLS assessment with TILs evaluation has been shown to improve prognostic accuracy. For example, BCa patients with moderate to abundant TLS in an invasive area but low-to-moderate TIL levels had worse outcomes than patients with high TILs densities [17]. In the same study [17], the authors observed that TILs percentages were significantly associated with adjacent TLS numbers and with higher disease-free survival (DFS) in HER-2/neu–positive but not in HER-2/neu–negative BCa. They also reported correlations between TLS grade and HER-2/neu protein expression or gene copy number, suggesting that recognition of immunogenic HER-2/neu epitopes by T cell clones, presented on mature DCs in the context of human leukocyte antigen (HLA)-A alleles, may drive T cell expansion and contribute to TLS formation. Berthe et al. [65] investigated the relationship between TLS score and intratumoral CD8^+^ T cells in NSCLC. They found that high densities of both CD8^+^ T cells and TLS were associated with a favorable prognosis; nevertheless, a substantial number of patients with good outcomes had high CD8^+^ T cell counts despite lacking TLS, indicating that CD8^+^ T cells alone may not reliably capture the prognostic value conferred by combined TILs/TLS assessment. Similarly, You et al. [66] reported that although high TILs levels often predict structurally mature and abundant TLS, some tumors exhibit high TILs without TLS formation, implying that additional factors influence TLS development.

**Figure 1:**
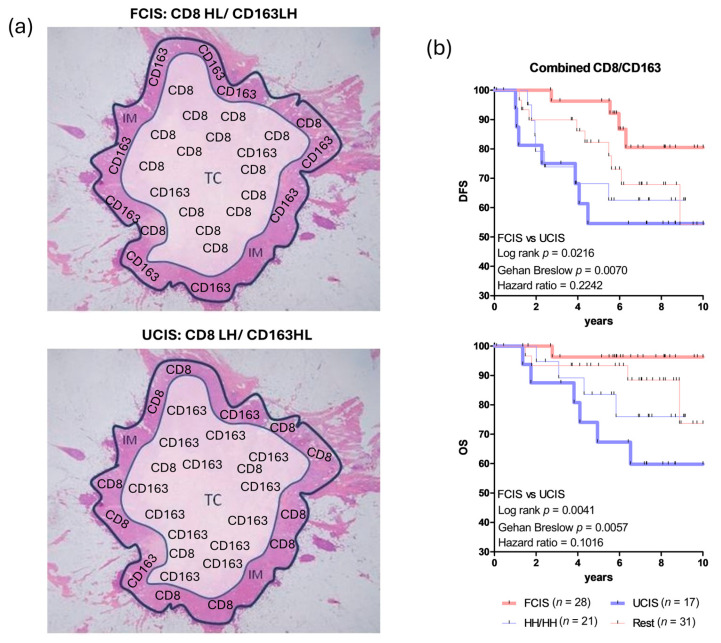
Joint assessment of CD8^+^ T cells and CD163^+^ macrophages intratumorally as prognostic biomarkers in breast cancer (BCa) patients. (**a**) FCIS (favorable combined immune signature): high CD8^+^ T cell density in the tumor center (TC) joined with low CD8^+^ T cell density in the invasive margin (IM) (group named CD8HL) in combination with low CD163^+^ macrophage density in TC joined with high CD163^+^ macrophage density in IM (group named CD163LH), altogether CD8HL/CD163LH. UCIS (unfavorable combined immune signature): low CD8^+^ T cell density in TC joined with high CD8^+^ T cell density in IM (group named CD8LH) in combination with high CD163^+^ macrophage density in TC joined with low CD163^+^ macrophage density in IM (group named CD163HL), altogether CD8LH/CD163HL. (**b**) Kaplan–Meier curves illustrating disease-free survival (DFS) and overall survival (OS) for BCa patients analyzed according to the joined densities of CD8^+^ T cells and CD163^+^ macrophages in the combined tumor regions TC and IM. FCIS and UCIS as in Figure 1a. HH/HH: high densities of CD8^+^ T cells analyzed in the combined tumor regions CT and IM/high densities of CD163^+^ macrophages analyzed in the combined tumor regions CT and IM; the Rest group includes: LL/LL (low densities of CD8^+^ T cells analyzed in the combined tumor regions CT and IM/low densities of CD163^+^ macrophages analyzed in the combined tumor regions CT and IM); HH/LL (high densities of CD8^+^ T cells analyzed in the combined tumor regions CT and IM/low densities of CD163^+^ macrophages analyzed in the combined tumor regions CT and IM); and LL/HH (low densities of CD8^+^ T cells analyzed in the combined tumor regions CT and IM/high densities of CD163^+^ macrophages analyzed in the combined tumor regions CT and IM). Statistically significant differences and hazard ratios between FCIS and UCIS groups are given. HH/HH, and Rest (LL/LL, HH/LL and LL/HH), could not significantly discriminate for DFS or OS. In fact, the LL/LL, HH/LL and LL/HH signatures showed a similar trend for improved clinical outcomes, albeit one inferior to that of FCIS; they were therefore grouped together as the “Rest” (Figure 1b is adapted from Ref. [67]).

In our earlier study, we tested whether quantifying CD8^+^ and CD163^+^ cells, individually and in combination, in both the tumor center (TC) and the invasive margin (IM) could refine immunoscoring in BCa and enhance conventional prognostic indicators [67]. We found that assessing cytotoxic CD8^+^ T cell and CD163^+^ suppressive macrophage densities in both TC and IM improved patient stratification and increased the prognostic accuracy of TNM staging. Moreover, a combined evaluation of these cell densities further enhanced predictions of DFS and overall survival (OS). Notably, the most favorable DFS and OS were observed when a high CD8^+^ T cell density in the TC was paired with a low CD8^+^ density at the IM (group CD8HL). By contrast, the opposite pattern, a low TC and high IM CD8^+^ density (CD8LH), was associated with higher recurrence rates and worse OS. For CD163^+^ macrophages, patients with low TC and high IM densities (CD163LH) had better outcomes, whereas those with high TC and low IM densities (CD163HL) fared worse. From these joint analyses, we defined favorable and unfavorable combined immune signatures for DFS and OS (FCIS and UCIS, respectively) (Figure 1a,b).

**Figure 2:**
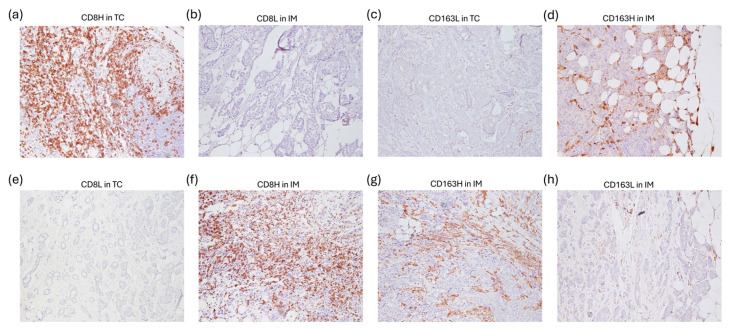
Images for intratumoral CD8^+^ T cell and CD163^+^ macrophage densities, comprising the combined immune signatures. (**a**–**d**) The favorable combined immune signature (FCIS) (CD8HL/CD163LH) in breast cancer (BCa) patients: (**a**) CD8 staining (100× magnification): high infiltration of CD8^+^ T cells in the tumor center (TC) (CD8H); (**b**) CD8 staining (200× magnification): invasive margin (IM) largely devoid of CD8^+^ T cells (CD8L); (**c**) CD163 staining (100× magnification): almost complete absence of CD163^+^ macrophages in TC (CD163L); (**d**) CD163 staining (200× magnification): high infiltration of CD163^+^ macrophages in IM (CD163H). (**e**–**h**) The unfavorable combined immune signature (UCIS) (CD8LH/CD163HL) in BCa patients: (**e**) CD8 staining (100× magnification): TC showing lack of CD8^+^ T cells (CD8L); (**f**) CD8 staining (200× magnification): IM densely populated by CD8^+^ T cells (CD8H); (**g**) CD163 staining (100× magnification): TC overpopulated by CD163^+^ macrophages (CD163H); and (**h**) CD163 staining (200× magnification): IM displaying minimal-to-absent CD163^+^ macrophages (CD163L).

Representative images of low and high CD8^+^ and CD163^+^ cell densities comprising the FCIS and UCIS are shown in Figure 2.

**Figure 3:**
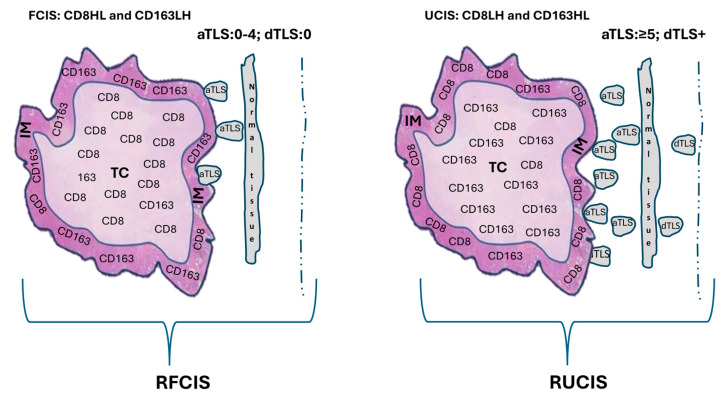
A schematic representation of intratumoral CD8^+^ T cells and CD163^+^ macrophages combined with tertiary lymphoid structures (TLS). The reinforced favorable combined immune signature (RFCIS) in breast cancer (BCa) patients comprises the favorable combined immune signature [FCIS: high and low densities of CD8^+^ T cells in the tumor center (TC) and invasive margin (IM), respectively, (CD8HL) combined with low and high densities of CD163^+^ macrophages in the same tumor regions (CD163LH)] joined with no adjacent TLS (aTLS) or up to 4 aTLS (aTLS:0–4) and no distal TLS (dTLS:0). The reinforced unfavorable combined immune signature (RUCIS) in BCa patients comprises the unfavorable combined immune signature [UCIS: low and high densities of CD8^+^ T-cells in TC and IM, respectively, (CD8LH) combined with high and low densities of CD163^+^ macrophages in the same tumor regions (CD163HL)] joined with 5 or more aTLS (aTLS: ≥5) and with distal TLS (dTLS^+^).

Approximately half of the patients in our cohort did not clearly fit into the favorable or unfavorable categories. Some exhibited high densities of both CD8^+^ and CD163^+^ cells in the TC and IM (HH/HH), while others displayed various combinations of these cell types across tumor compartments (the “Rest” group) (Figure 1b). This highlighted the need for an additional, sensitive biomarker within the TME to better classify these patients. To improve the prognostic utility and broader applicability of FCIS and UCIS, we re-examined our cohort by integrating peritumoral TLS patterns. This produced two new composite signatures: the reinforced favorable combined immune signature (RFCIS) and the reinforced unfavorable combined immune signature (RUCIS), which combine the FCIS or UCIS with the number and location of peritumoral TLS [adjacent TLS (aTLS) versus distal TLS (dTLS)] (Figure 3) [20].

**Figure 4:**
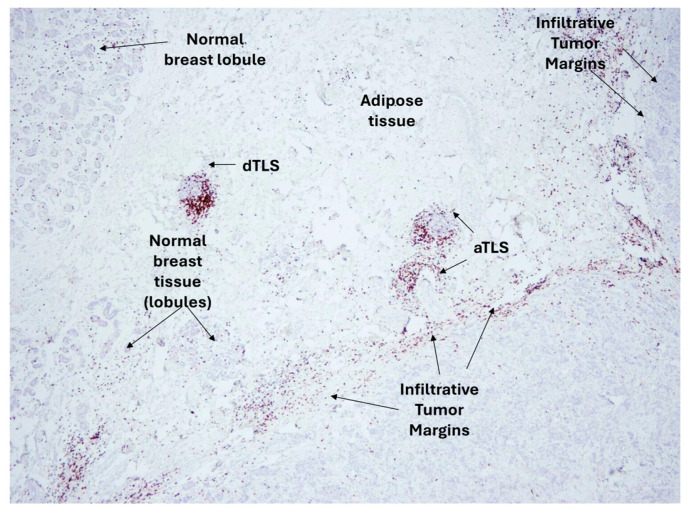
Representative images of adjacent tertiary lymphoid structures (aTLS) and distal TLS (dTLS). Microscopy images from invasive ductal breast cancer sections (adapted from Ref. [20]).

Patients in the RFCIS group were CD8HL and CD163LH (i.e., FCIS), had no or up to four adjacent TLS (aTLS: 0–4), and no distal TLS (dTLS: 0). Patients in the RUCIS group were CD8LH and CD163HL (i.e., UCIS), had five or more adjacent TLS (aTLS: ≥5), and, additionally, harbored distal TLS (dTLS^+^). Representative microscopy images of aTLS and dTLS in invasive ductal breast cancer are shown in Figure 4.

**Figure 5:**
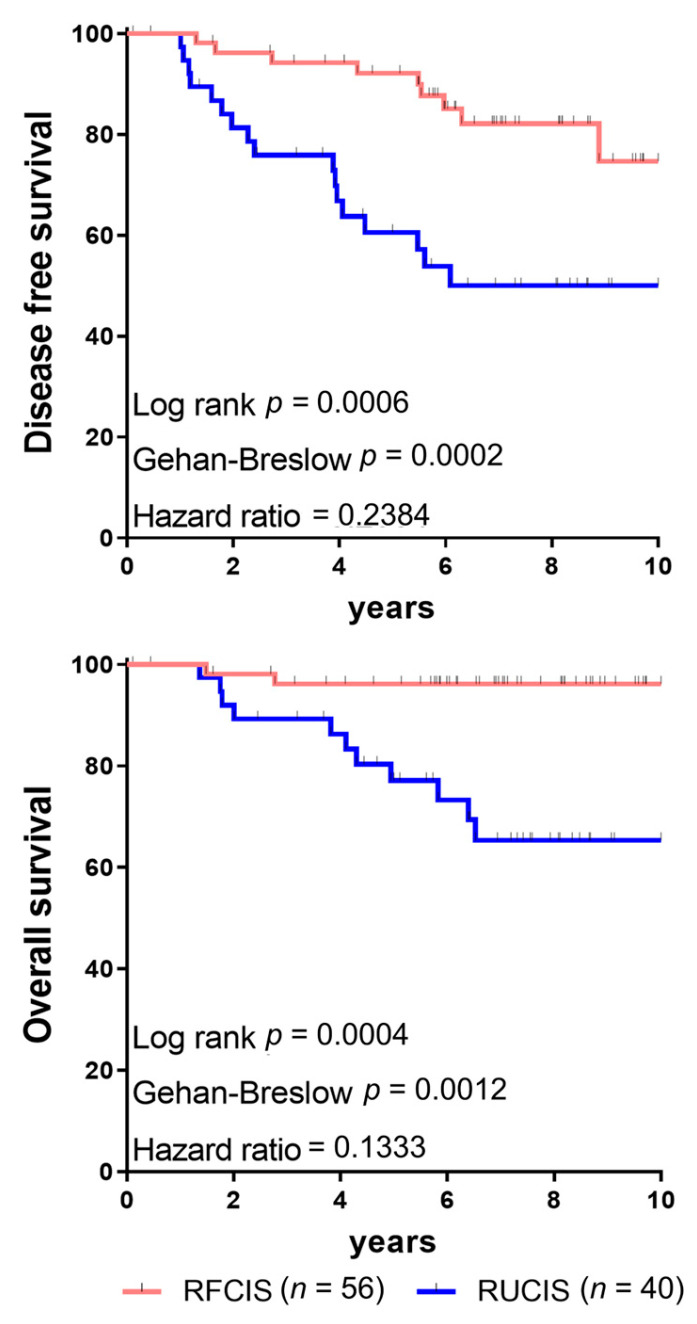
Clinical outcomes in breast cancer (BCa) patients. Disease-free survival and overall survival in BCa patients with reinforced favorable combined immune signature (RFCIS) or reinforced unfavorable combined immune signature (RUCIS) (adapted from Ref. [20]).

BCa patients classified as RFCIS, regardless of molecular subtype, had significantly better DFS and OS than those in the RUCIS group (Figure 5). We also observed that tumors with high numbers of dTLS were infiltrated by greater frequencies of FoxP3^+^ Tregs and correlated with poorer outcomes in patients with invasive ductal adenocarcinoma of the breast [20].

These findings, together with reports linking peritumoral TLS to unfavorable prognoses, led us to hypothesize that immune elements within the IM or peritumoral regions may signal or facilitate malignant cell migration beyond the tumor margin, thereby promoting invasion and metastasis. Supporting this idea, Liu et al. [18] observed cancer-cell clusters infiltrating peritumoral TLS that resemble those seen in lymph node metastases, suggesting that tumor cells within TLS may have an increased propensity to spread to regional nodes. In similar lines, extratumoral TLS in patients with colorectal cancer were associated with more advanced tumors, implying their development in response to ongoing progression [24]. Also, the study by Finkin et al. [7] showed that inflammation-associated ectopic TLS can act as immune-dependent tumor microniches that potentially contribute to recurrence in hepatocellular carcinoma, attributing a poor prognostic role to peritumoral TLS, despite generally positive associations with intratumoral TLS in that disease. Thus, peripheral TLS may foster an immunosuppressive microenvironment that supports tumor growth, either by inhibiting antitumor immunity or by directly facilitating tumor progression and dissemination.

Taken together, integrating spatially resolved immune-cell densities with precise mapping of peritumoral TLS would likely provide a more powerful and biologically informative prognostic framework than the quantification of TILs alone: RFCIS identifies patients with markedly superior DFS and OS, whereas RUCIS marks those at higher risk. The contrasting prognostic roles of intratumoral versus peripheral TLS, along with the enrichment of FoxP3+ Tregs and evidence of tumor cell involvement in distal TLS, suggest that the location and maturity of TLS critically determine whether they support antitumor immunity or paradoxically promote immune suppression and dissemination. These observations underscore the need for mechanistic studies to dissect how TLS composition and spatial relationships with tumor and stromal compartments drive divergent outcomes, and they argue for the standardization of TLS/TIL scoring and the prospective, multicenter validation of RFCIS/RUCIS. Ultimately, a validated, spatially informed immune classifier could guide patient stratification and inform rational combinations of immunotherapy, as well as macrophage-targeting or TLS-modulating strategies to convert unfavorable microenvironments into durable antitumor niches.

## 5. Chemokine-Driven TLS: Enhancing Antitumor Immunity and Synergy with Checkpoint Inhibitors

Chemokines play a crucial role in the well-documented ability of TLS to effectively attract and accumulate specific immune cell populations, as well as in their established involvement in the development and operation of peripheral lymph nodes [5,6]. Understanding the prognostic and therapeutic implications of TLS is therefore important, and chemokine-based strategies to promote TLS formation and maturation are being explored to enhance antitumor immunity. Furthermore, ICIs or therapeutic vaccines may preserve TLS functionality, supporting the generation and persistence of endogenous antitumor responses that begin locally and can expand systemically, ultimately improving OS.

Chemokines coordinate the recruitment and migration of lymphocytes and DCs into tissues, enabling cell–cell interactions and mutual activation which are essential for forming TLS and fully developed HEVs. The presence of mature FDCs may contribute to HEV initiation and maintenance within TLS [68]. Dieu-Nosjean et al. [49] reported that T-cell infiltration of TLS via HEVs and their subsequent activation are governed by a complex chemokine signature, opening new avenues to refine and potentiate cancer immunotherapy. A recent comprehensive review similarly describes how coordinated homeostatic chemokines regulate lymphocyte entry through HEVs into TLS and their activation, underscoring translational opportunities for cancer treatment [69]. Mature DCs within TLS play a prominent role in activating T cells by presenting tumor peptides, thereby generating tumor-specific immune memory that can be reactivated and amplified during ICI therapy [15,70,71]. In this context, chemokines are key regulators of the pathways that potentiate antitumor immunity within TLS. Messina et al. [72] identified a 12-chemokine gene signature associated with the presence of TLS in melanoma metastases and with improved clinical outcomes. These TLS were built from the essential elements of the immune system, incorporating lymphatic channels, DCs and other antigen-presenting cells, and clusters of B cells positioned next to CD8^+^ and CD4^+^ T-cell areas. Notably, FoxP3^+^ Tregs were sparse, and the 12-chemokine signature did not include chemokines known to specifically recruit or sustain Tregs, such as CCL1, CCL20, or CCL22 [73,74]. In an analysis of 83 metastatic melanoma specimens, Karapetyan et al. [75] found that TLS with GCs predominantly resided in peritumoral or stromal niches. Importantly, the previously defined 12-chemokine signature [72] was significantly enriched in samples with TLS versus those without, and the signature-positive group exhibited superior overall survival. Along the same lines, the expression of specific chemokines and chemokine receptors have been examined in various cancers.

In NSCLC, ovarian and nasopharyngeal cancers, CXCL13-producing CD4^+^ T cells within the TME are predominantly localized at the core of TLS, display a Tfh cell phenotype, and function as potentiators of antitumor immunity [76,77,78]. Notably, CXCL13 has been recognized as one of the most reliable biomarkers, predicting improved survival across multiple human cancers [77,78]. Its production by infiltrating CD4^+^ Tfh cells within inflamed tissues is considered a critical initiating event in TLS formation and maintenance as well as in the establishment of dynamic GCs, which are critical for initiating and shaping antitumor immune responses [76]. Furthermore, CXCL13 expression is closely associated with the recruitment of CXCR5^+^ immune cells to tumor sites, directing and positioning them within lymphoid follicles, particularly CXCR5^+^ B cells [79]. Ding et al. [80] showed that CXCL13-dependent recruitment of CXCR5^+^ B cells into TLS located within or adjacent to the TME plays a crucial role in presenting tumor antigens to Tfh and CXCL13^+^ CD8^+^ cytotoxic T cells in melanoma patients receiving ICI therapy, thereby improving survival probability. Metastatic sites that harbor CXCR5^+^ B cells have also been implicated in activating neoantigen-specific CXCL13^+^ CD4^+^ and CXCL13^+^ CD8^+^ T cells that predict responses to ICIs [81,82]. The high expression of CXCL13 by both CD4^+^ and CD8^+^ T cells, many of which bear receptors that recognize neoantigens, and their likely cooperation with CXCR5^+^ B cells suggests that the activation of neoantigen-specific TILs may occur within TLS found in metastatic deposits.

**Figure 6:**
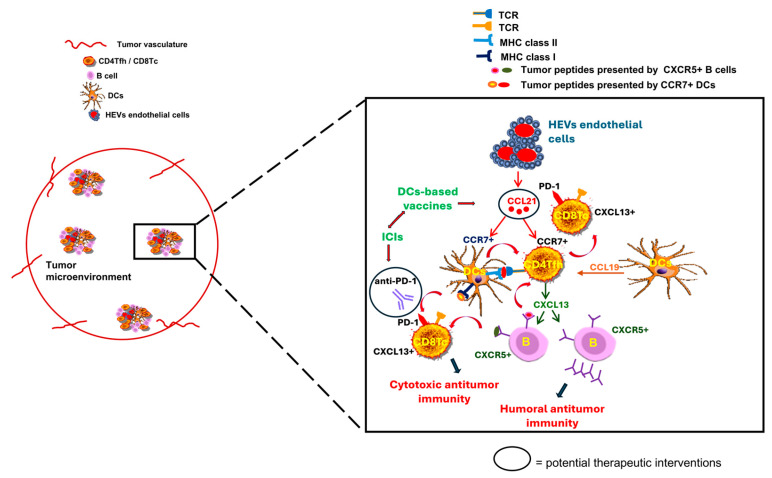
Tertiary lymphoid structure (TLS) composition, formation, and modulation. CCR7^+^ CD4^+^ T cells within the tumor microenvironment (TME) display a T follicular helper (Tfh) phenotype and secrete CXCL13, a key chemokine involved in TLS formation and maintenance. CXCL13-dependent recruitment of CXCR5^+^ B cells into TLS in close proximity to or within the TME plays a crucial role in presenting tumor antigens to CD4^+^ Tfh and CD8^+^ cytotoxic T cells (CD8^+^ Tc). This process supports the establishment of dynamic germinal centers (GCs), which serve as crucial sites for initiating and shaping antitumor immune responses: CD8^+^ Tc expressing programmed cell death protein-1 (PD-1) can be specifically targeted by immune checkpoint inhibitors (ICIs) for generating a more potent cytotoxic antitumor immunity. CXCL13-activated B cells have enhanced production of antibodies against tumor antigens, also resulting in increased humoral antitumor immunity. CD4^+^ Tfh and CD8^+^ T cells can be activated by CCR7^+^ dendritic cells (DCs) via major histocompatibility complex (MHC)-restricted presentation of antigenic tumor peptides. Specific immune signaling chemokines, including CCL19 and CCL21, mainly produced by DCs and high endothelial venules (HEVs) endothelial cells, appear to be crucial for the development of TLS: CCL21 produced by HEVs endothelial cells activate both CCR7^+^ DCs and CCR7^+^ CD4^+^ Tfh cells, whereas CCL19 produced by activated DCs further promotes the capacity of CD4^+^ Tfh cells to support the cytotoxic function of CD8^+^ Tc. Therapeutic targeting of CCL21 via genetically engineered DCs-based vaccines alone or combined with ICIs has shown promising antitumor efficacy.

Additional chemokines implicated in TLS development include CCL19 and CCL21, predominantly produced by DCs and HEV endothelial cells; their upregulation in tumor-associated TLS has prompted interest in their use as biomarkers and as targets for therapeutic interventions [13,31,35]. When produced within the TME, these chemokines recruit CCR7^+^ CD4^+^ Tfh cells and CCR7^+^ DCs, thereby promoting TLS formation and strengthening antitumor immunity [13,49]. Emerging evidence indicates that enhancing CCL21 or CCL19 gradients within tumors can markedly boost anti-PD-1 efficacy. By recruiting and organizing T cells and DCs into TLS-like niches, these chemokines promote local antigen presentation and sustain cytotoxic T cell priming [83,84,85]. The therapeutic delivery or induction of CCL21/CCL19 in tumors increases intratumoral CD8^+^ T cell infiltration and induces adaptive PD-L1 upregulation, changes that render the TME more responsive to PD-1 blockade [83,84,86]. Accordingly, strategies that raise intratumoral CCL19/CCL21 concentrations levels, either via engineered DCs-based vaccines or gene therapy, have the potential to convert immunologically “cold” tumors into inflamed sites and improve responses to anti-PD-1 therapy. For example, intratumoral delivery of CCL21-modified DCs increased intratumoral CD8^+^ T cell frequencies, and upregulated PD-L1 expression and systemic immune activation in NSCLC, providing a strong rationale for combining CCL21-based vaccines with PD-1 blockade [87]. Similarly, higher intratumoral CCL19^+^ DCs signatures correlated with CCR7^+^ CD4^+^ Tfh cell-dependent activation of cytotoxic CD8^+^ T cells with better responses to PD-L1 inhibitors in TNBC, suggesting that targeting CCL19 could prime tumors for checkpoint therapy [12,85]. Preclinical and early-phase clinical studies further show that CCL21-DCs tumor-lysate vaccines can augment anti-PD-1 activity, underscoring the broader potential of modulating TLS-associated chemokines [86,88,89,90,91]. Vaccine-based delivery of CCL21 may be particularly useful for tumors that cannot receive direct intratumoral injections or require repeated administrations. Moreover, pairing CCL21 with PD-1 blockade could extend the benefits to patients who do not respond to PD-1 inhibitors alone. Figure 6 depicts the intra-TLS cellular organization and interactions based on chemokine/chemokine-receptor axes in the context of immunotherapies, including ICIs and DCs-based vaccines.

Beyond chemokines, inflammatory cytokines such as lymphotoxin, IL-17, IL-22, and IL-23 have been proposed to promote TLS formation by driving Tfh cell differentiation, which in turn supports B cell activation and maturation. Thus, therapeutic strategies that use chemokines, cytokines, or their agonists may offer a promising approach to induce TLS formation and maturation in cancer, with the goal of potentiating endogenous antitumor immunity and improving outcomes for patients treated with ICIs.

Collectively, chemokines emerge as central architects of TLS, orchestrating the recruitment, positioning, and activation of lymphocytes and DCs that underpin GCs activity and durable antitumor immunity. CXCL13 stands out both as a mechanistic driver of TLS formation and a robust biomarker of improved survival, while CCL19/CCL21 gradients, when enhanced by engineered DCs, vaccines, or gene-delivery approaches, offer a practical route to build TLS-like niches, increase intratumoral T cell priming and sensitize previously “cold” tumors to PD-1/PD-L1 blockade. Preclinical and early clinical evidence thus supports chemokine-based strategies, alone or paired with ICIs or vaccines, to potentiate local antigen presentation and systemic immune responses; however, realizing this potential will require rigorous biomarker-driven patient selection, optimized delivery platforms, and careful assessment of safety and durability. Ultimately, translational efforts that precisely induce and sustain mature, non-suppressive TLS could convert mechanistic insight into tangible clinical benefits, improving responses to immunotherapy and patient outcomes.

## 6. TLS as Predictive Biomarkers for Tailored Cancer Immunotherapy

High expression of immune checkpoint molecules has been observed in tumors with abundant TILs infiltration and numerous TLS [92], further supporting the role of TLS in eliciting immune responses. Within the follicular B cell zones of TLS, PD-1-expressing CD8^+^ as well as Tfh CD4^+^ T cells and DCs are present, highlighting TLS as sites that coordinate T–B cell interactions during immunotherapy and thereby drive antitumor immunity [4,93]. The function of TLS can be modulated by ICIs and other immunotherapies, which provides a rationale for combination treatment approaches. Voabil et al. [94] found that the TME of patients who responded to ICI therapy contained numerous large TLS infiltrated by dense populations of PD-1high CD39^+^ CD103^+^ memory T cells. Helmink et al. [61] recently provided evidence that B cells within TLS are key mediators of ICI responses in metastatic melanoma and renal cell carcinoma. Although the precise mechanisms remain incompletely defined, their findings suggest that the beneficial functions of memory B cells and plasma cells, critical for adaptive immunity, may include the enhancement of T cell responses following ICIs therapy. Importantly, they identified memory plasma B cells in ICI responders, implying that these cells may contribute to antitumor activity by producing tumor-targeting antibodies. Cabrita et al. [95] analyzed samples from patients with metastatic melanoma and found that tumors containing TLS infiltrated by both CD8^+^ T cells and CD20^+^ B cells were associated with improved survival, independent of other clinical factors. They also observed that B-cell-rich tumors harbored higher numbers of naïve and/or memory T cells, and they identified a TLS-associated gene-expression signature that predicted clinical outcomes in patients treated with ICIs, independently of tumor mutational burden. Hayashi et al. [96] reported that tumors with a high density of mature TLS were linked to earlier disease stage and longer survival in esophageal squamous cell carcinoma, and that TLS predicted both response to anti-PD-1 therapy and OS in these patients. In soft-tissue sarcomas, a distinct TLS profile characterized by active CD8^+^ T cells, PD-1 expression, and prominent B cell infiltration was associated with better clinical outcomes and identified a subgroup of patients who showed improved survival from PD-1 inhibitors [97]. In the PEMBROSARC phase II trial, patients with sarcomas enriched for TLS showed significantly higher response rates to ICIs and longer DFS than the overall cohort [98]. These studies proposed TLS as a key biomarker for selecting soft-tissue sarcoma patients likely to benefit from ICIs.

In melanoma models, TLS appear to help maintain an immune-active TME by fostering interactions among B cells, T cells, and other critical immune components. Initial murine studies demonstrated that ICIs therapy promotes the development and organization of tumor-associated TLS and correlates with reduced tumor volume [99]. In B16-OVA melanoma grafts in C57BL/6 mice, anti-PD-1 and combined anti-CTLA-4/anti-PD-1 treatments produced larger and more numerous TLS with distinct T- and B-cell zones; TLS presence, number, and size correlated with a reduced tumor burden and improved response to checkpoint blockade [61,99,100]. In intraperitoneal B16-OVA models, PD-1 or CTLA-4 blockade induced organized TLS formation, driven by a cancer-associated fibroblast subset acting as a lymphoid-tissue organizer upon checkpoint inhibition [100,101]. Lynch et al. [102] reported that nearly half of cutaneous metastatic melanoma lesions contained TLS, predominantly in the peritumoral region. TLS presence correlated with increased intratumoral lymphocyte infiltration, although the functional activity of lymphocytes within TLS differed from that inside the tumor. Multivariate hazard models adjusting for surgical intent and intratumoral CD8^+^ T cell activity showed that TLS presence was associated with improved OS. Moreover, intra-TLS B cell activity strongly correlated with survival: a high frequency of B cells undergoing somatic hypermutation was associated with better outcomes, whereas CD21-expressing B cells correlated with a worse prognosis. These findings indicate that each TLS constitutes a distinct immunological niche separate from the tumor itself, and that the way this niche shapes B cell function is critical for systemic tumor control.

In the setting of neoadjuvant immunotherapy, TLS have also emerged as robust prognostic biomarkers. Patients receiving neoadjuvant chemoimmunotherapy showed an increase in both the number and maturity of TLS, with the degree of TLS maturation proving more informative than mere abundance for postoperative risk assessment in resectable NSCLC cases [103]. Moreover, in high-risk urothelial carcinoma patients treated with combined PD-L1 and CTLA-4 inhibition, a TLS-linked eight-gene signature was more highly expressed in responders than in non-responders, reinforcing the promise of TLS for identifying those patients most likely to benefit from ICI therapy [104].

Therapeutic vaccination in tumor patients can also trigger TLS formation with improved clinical outcomes. Melssen et al. [105] examined the TME of patients with resected, high-risk melanoma who received either a multipeptide vaccine in incomplete Freund’s adjuvant or recombinant MAGE-A3 delivered with AS15 (an adjuvant containing TLR4 and TLR9 agonists). Both vaccine regimens promoted the infiltration of adaptive and innate immune cells and produced sustained upregulation of PD-L1 and IDO-1 (indoleamine 2,3-dioxygenase 1), indicating concurrent immune activation and counter-regulatory signaling in the TME. The authors also observed clear intratumoral formation of TLS and increased expression of TLS-related genes, changes that were associated with systemic immune responses and clinical outcomes. In a separate vaccine study [106], patients with pancreatic ductal adenocarcinoma (PDAC) received an irradiated, allogeneic pancreatic ductal adenocarcinoma cell vaccine engineered to secrete granulocyte-macrophage colony-stimulating factor (GM-CSF), namely GVAX, either alone or combined with low-dose cyclophosphamide to selectively deplete Tregs. An analysis of the resected tumors two weeks after vaccination revealed that most patients developed intratumoral tertiary lymphoid aggregates induced by the vaccine. Immunohistochemistry characterized these aggregates as organized, regulatory hubs of adaptive immunity. Microdissection followed by microarray profiling of the aggregates uncovered gene-expression signatures across five signaling pathways linked to immune-cell activation and trafficking; these signatures correlated with improved post-vaccine immune responses. Within the aggregates, the suppression of Treg-associated pathways and upregulation of Th17 pathways were linked with longer survival and higher intratumoral Teffector–Treg ratios. This study provided a direct demonstration that vaccine therapy can convert a “non-immunogenic” PDAC into an “immunogenic” tumor by driving T cell infiltration and the formation of TLS. Notably, the post-GVAX tumor infiltration and aggregate formation also triggered counter-regulatory mechanisms, including increased PD-1/PD-L1 signaling, implying that vaccine-primed PDAC might be more responsive to subsequent immune-checkpoint therapies than vaccine-naïve tumors. TLS formation and clonal expansion have also been observed in regressing lesions of patients with high-grade cervical intraepithelial neoplasia (CIN2/3) after vaccination against human papillomavirus oncoproteins [107]. Post-vaccination cervical stroma showed organized tertiary lymphoid-like structures under the remaining intraepithelial lesions and, in contrast to unvaccinated lesions, contained antigen-driven proliferating immune cells. Molecularly, these stromal changes involved an increased expression of immune activation and effector genes and coincided with an immunologic signature and T cell clonal expansion [107]. In a murine glioblastoma model, vaccination with the human cytomegalovirus (HCMV) immediate-early protein 1 (IE1) induced pronounced tumor regression and extended survival; these outcomes correlated with a greater number and larger size of TLS compared to control animals. TLS presence was linked to an elevated T-cell infiltration, an expansion of effector-memory T cells, and a substantial population of activated B cells in the draining lymph nodes [108].

We should also consider that presence, frequency, and cellular makeup vary between cancers and appear to influence both clinical outcomes and responses to immunotherapy. Importantly, not only the quantity but also the anatomical location and immune composition of TLS determine whether their impact is beneficial or detrimental. Across several malignancies, including breast, colorectal, and gastric cancers, patients whose tumors harbor a high density of TLS show greater immune cell infiltration and, correspondingly, an improved prognosis and better responses to immunotherapy when compared with patients with few TLS [109,110,111]. In squamous cell carcinoma of the lung, TLS abundance has emerged as an independent prognostic indicator that can outperform conventional staging in chemotherapy-naïve cohorts [112,113]. In hepatocellular carcinoma, composite metrics that combine TLS density with systemic inflammation markers yield stronger prognostic information than TLS counts alone: specifically, the concurrence of abundant TLS and a low neutrophil-to-lymphocyte ratio in peripheral blood more accurately reflects intratumoral immune status and predicts survival [114]. Conversely, another HCC study reported that increased peritumoral TLS correlated with greater neutrophil infiltration and worse outcomes [115]. A spatially dependent effect of TLS has also been observed in cholangiocarcinoma, colorectal cancer with liver metastases, and clear cell renal carcinoma: intratumoral TLS are generally associated with favorable survival, whereas a high burden of peritumoral TLS correlates with a poor prognosis. Multiplex immunohistochemistry has begun to explain these opposing associations; intratumoral TLS tend to be functionally mature, enriched for Tfh cells, and supportive of effective antitumor immunity, while peritumoral TLS often appear rudimentary and dysfunctional, dominated by regulatory T cells and M2 macrophages and lacking Tfh. Moreover, increases in peritumoral TLS have been linked to a relative rise in Treg proportions within intratumoral TLS in some datasets [116,117,118]. Taken together, these findings underscore that both the location and cellular composition of TLS are critical determinants of their prognostic significance during immunotherapies, arguing for spatially resolved and multi-parameter immune profiling in future TLS studies. Table 1 provides a brief summary of the aforementioned studies that highlight the important role of TLS as a prognostic biomarker for immunotherapies in various types of cancer.

In summary, a growing body of preclinical and clinical evidence positions TLS as central organizers of local antitumor immunity and as important determinants of responses to immunotherapies and therapeutic vaccination. The presence, maturity, and cellular composition of TLS correlate with improved clinical outcomes across multiple tumor types and appear inducible by checkpoint blockade and vaccine strategies. These observations highlight TLS both as predictive biomarkers to refine patient selection for immune-based therapies and as manipulable microanatomical platforms for combination approaches aimed at converting immunologically “cold” tumors into responsive, immune-active lesions. Moving forward, harmonized methods to quantify TLS, mechanistic studies to dissect how distinct TLS niches shape B- and T-cell function, and future clinical trials that prospectively integrate TLS-directed interventions will be essential to translate these insights into more durable, broadly effective cancer immunotherapies.

## 7. The Critical Contribution of CD4^+^ T Cells to the Development of Robust Antitumor CD8^+^ T Cell Responses

**Figure 7:**
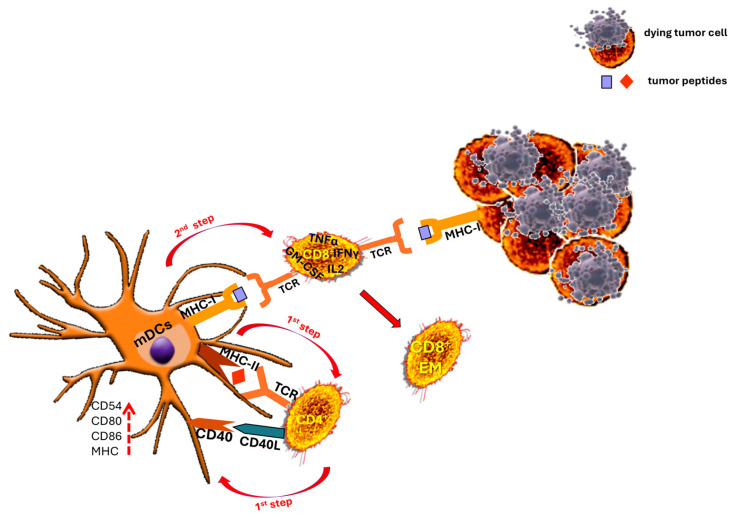
Tertiary CD4^+^ T-cell-dependent dendritic cell (DCs) maturation is essential for robust CD8^+^ T-cell-mediated lysis of autologous tumor cells. First Step: DCs–CD4^+^ T cell crosstalk and DCs maturation. DCs uptake tumor antigens and display tumor-derived peptides on major histocompatibility complex (MHC) class II molecules. Naïve CD4^+^ T cells recognize these peptide–MHC class II complexes with their T cell receptors (TCR); antigen recognition activates CD4^+^ T cells, which in turn express CD40 ligand (CD40L). Engagement of CD40L with CD40 on DCs provides a potent maturation signal for the DCs. Matured DCs (mDCs) acquire a phenotype, upregulating MHC class II (HLA-DR) molecules, costimulatory proteins (CD80, CD86), and adhesion molecules such as CD54, so that both partners (CD4^+^ T cells and DCs) acquire reinforced activation and functional competence. Second Step: mDCs drive CD8^+^ T effector cell differentiation and tumor killing. mDCs present tumor peptides on MHC class I to CD8^+^ T cells, supplying antigenic signals plus enhanced co-stimulation and cytokine support. This drives strong CD8^+^ T cell activation that produce effector cytokines (for example IL-2, IFN-γ, TNF-α, and GM-CSF) and acquire efficient lytic machinery. Those CD8^+^ T cell effectors recognize the identical tumor peptides when presented on tumor cell MHC class I molecules and mediate antigen-specific killing of autologous tumor cells.

Beyond direct induction through immunotherapy, TLS can enhance antitumor CD8^+^ T cell activity through multiple mechanisms. In a large single-cell spatial analysis, Kasikova et al. [119] linked TLS maturation to enriched helper T cell programs and to the recruitment and activation of antigen-reactive CD8^+^ T cells, highlighting the cooperative interactions among CD4^+^ T cell subsets, B cells, and DCs within TLS. Similarly, Liu et al. [120] showed that CD4-lineage signatures (including Tfh-like cells and CXCL13 producers) are concentrated in TLS microdomains that spatially correlate with CD8^+^ T cell infiltration and activation signatures. Other studies found that the DC subsets present in TLS are required for local antigen presentation and CD8^+^ T cell priming, emphasizing CD4^+^ T-cell-mediated DCs-licensing within TLS as a key pathway for generating functional CD8^+^ effectors [121,122]. In a pancreatic cancer model, the activation of MHC class II-restricted CD4^+^ T cells by a neoantigen vaccine, improved the priming, recruitment, infiltration, and effector functions of neoantigen-specific CD8^+^ T cells, resulting in enhanced antitumor reactivity [123]. These findings align with our earlier observations [50] of the essential role of CD4^+^ T-cell-activated DCs for optimal CD8^+^ T-cell-mediated killing of autologous tumors based on three cell-type interactions in two steps: in the first step, DCs activate CD4^+^ T cells by presenting the tumor peptides in the context of their MHC class II molecules. CD4^+^ T cells recognize this peptide via their specific receptors and once activated, they generate mature ”licensed” DCs (mDCs) via CD40 ligand (CD40L)–CD40 interactions. These mDCs express increased levels of costimulatory proteins (CD80, CD86), adhesion molecules (ICAM-1/CD54), and MHC class II (HLA-DR) molecules; thus, in this first step, mutual activation between CD4^+^ T cells and DCs occurs. In the second step, the mDCs activate the CD8^+^ T cells presenting tumor peptides via their MHC class I molecules. The resulting hyper-activated CD8^+^ T cells produce IL-2, interferon (IFN)γ, tumor necrosis factor (TNF)α, and GM-CSF and efficiently lyse autologous tumor cells via recognition of the same peptides this time presented to them in the context of the tumor’s MHC class I molecules (Figure 7).

Collectively, these data underscore the indispensable role of CD4^+^ T cells in mounting cytotoxic responses against patient-derived tumors. Because malignant cells typically lack surface MHC class II expression, CD4^+^ T cells can only recognize tumor antigens after cross-priming by autologous DCs [124,125]. Thus, tumor-specific CD4^+^ T cells must be present at the sites of antigen presentation, such as the tumor-draining lymph nodes, to expand the pool of activated cytotoxic T cells. We propose that, within TLS, mDCs present tumor-associated antigens and sustain the local generation of antigen-specific T cells directly within the TME. Consequently, TLS can maintain adaptive, localized immune responses that evolve with the tumor’s antigenic landscape, making them privileged niches for T cell recruitment and activation at the primary tumor site and likely contributors to improved patient survival. Overall, lymphoid neogenesis within tumors appears to be a critical driver of protective immune responses across multiple cancer types and offers promising avenues for advancing immunotherapeutic strategies.

## 8. Conclusions

Cancer immunotherapy is rapidly changing with regard to how we predict treatment responses. Recent evidence positions TLS as emerging biomarkers that may offer superior prognostic and predictive value compared to PD-L1 expression and standard TIL enumeration. In the context of anti-PD-1/PD-L1 therapies, TLS have shown important value in distinguishing responders from non-responders by reshaping the TME. The biology of TLS is complex and multifaceted, offering both opportunities and challenges. Their diverse roles and mechanisms of formation can profoundly influence tumor progression and patient outcomes, indicating that TLS could meaningfully advance therapeutic strategies. At the same time, our understanding remains incomplete: more work is needed to define how ICIs-based treatments affect adaptive immune populations, especially B and T cells, and to clarify the interactions between TLS and CD8^+^ T cells. Such insights could guide the selection of therapeutic combinations that pair B-cell-directed approaches with existing T-cell-focused immunotherapies.

Our review brings together mechanistic, spatial, and clinical evidence with a distinctive emphasis on integrating both T- and B-cell biology into a unified framework for TLS assessment. We highlight practical avenues for standardizing TLS evaluation and provide a translational roadmap that links TLS composition to trial design and biomarker development. By underscoring where the evidence base is strongest, where gaps persist, and which measurements hold the greatest promise for improving patient stratification, this review aims to advance TLS-informed immuno-oncology.

Beyond their use as biomarkers, TLS themselves are attractive therapeutic targets. Standardized pathology methods are required to detect and quantify TLS reliably, since retrospective data suggest that TLS may predict clinical responses better than PD-L1 expression in some early-stage settings. Prospective studies are essential to determine how TLS spatial distribution and maturity influence prognosis. Moreover, strategies to induce TLS formation, likely through ICIs, targeted chemokine or cytokine delivery, or therapeutic vaccination, show promise, but we must decode the cellular and molecular mechanisms that determine why some patients respond while others do not.

Both T-cell- and B-cell-driven mechanisms are essential for TLS function and antitumor immunity, and an exclusive focus on T cells risks overlooking important B-cell contributions including GCs reactions, antigen presentation by B cells, affinity-matured tumor-specific antibodies, and local plasma and memory B cells that shape and sustain effective responses. To provide a more balanced and clinically useful portrait of TLS biology, future diagnostic and mechanistic studies should routinely incorporate B-cell-focused readouts into TLS scoring and analysis; for example, the following could be employed: CD20 and CD21 staining to define B-cell follicles and follicular DCs networks; markers of GCs activity such as BCL6 or direct evidence of somatic hypermutation; plasmablast/plasma cell quantification (e.g., CD38, CD27, CD138); assessment of antibody clonality/affinity and tumor-specific immunoglobulin features; and spatial analyses that capture B–T interactions. Embedding these measures alongside T-cell metrics will produce more comprehensive, prognostically and predictively powerful TLS assessments and better inform immunotherapeutic strategies.

The prospect of deliberately promoting TLS formation and maturation to improve outcomes is an exciting frontier. Early preclinical results are encouraging, but rigorous clinical trials are needed to evaluate the benefit of integrating TLS- or B-cell-targeted interventions with current immunotherapies. Advances in multiplexed and spatial technologies will deepen our understanding of TME and TLS composition, revealing the cellular and molecular events that underpin successful antitumor immunity and enable more effective, personalized cancer treatments.

## Figures and Tables

**Table 1:** Summary of the studies highlighting the predictive role of TLS in cancer immunotherapy.

Location	Findings/Outcomes	Reference(s)
TLS in TME	High frequencies of CD8^+^ PD-1^+^, cytokine-producing Tfh CD4^+^ cells, and DCs within TLS B-cell zones generating antitumor mechanistic pathways during immunotherapy.	[4,92,93]
	High frequencies of TLS-associated PD1^+^ CD39^+^ CD103^+^ T memory cells correlate with responses to anti-PD-1.	[94]
	Memory B cells within TLS regulate clinical outcomes with ICIs in melanoma and renal cell carcinoma patients.	[61]
	TLS infiltrated by high numbers of TILs (CD8^+^, CD20^+^, T_naive_, T_memory_) and expressing an activated gene signature correlate with favorable clinical responses with ICIs in melanoma.	[95]
	High density of mature TLS predicted responses to anti-PD-1 and prolonged OS.	[96]
	Favorable clinical outcomes with PD-1 inhibitors in patients with soft tissue sarcomas having TLS with strong infiltration by CD8^+^ cells, B cells, and high PD-1 expression.	[97,98]
	Favorable response to neoadjuvant chemoimmunotherapy in NSCLC in the presence of high numbers mature TLS.	[103]
	Favorable response to neoadjuvant immunotherapy I urothelial carcinoma in the presence of TLS expressing high levels of an 8-effector gene signature.	[104]
	High densities of TLS expressing a 9-gene signature. Favorable clinical outcomes in breast cancer.	[109]
	High average level of TLS abundance predict favorable clinical responses in HCC, GC, colon cancer, and squamous lung cancer.	[110,111,112,113]
TLS in peritumoral regions of melanoma metastatic lesions	Improved clinical outcomes with TLS-infiltrated B cells possessing Ig somatic hypermutations and worse outcomes associated with high densities of CD21^+^ B cells.	[102]
TLS peritumoral	Increased DFS in breast and NSCLC patients associated with high TLS densities.	[20,114]
	Increased TLS densities associated with progressively worse outcomes in HCC.	[115]
	High peritumoral TLS density positively correlated with unfavorable clinical responses whereas abundant intratumoral TLS density positively correlated with increased survival in CCA as well as in ccRCC and metastatic colorectal cancer.	[116,117,118]
TLS in TME (vaccine-induced)	Generation of TLS with high PD-1^+^, IDO^+^ expression upon vaccination with a multivalent vaccine or with recombinant MAGE in melanoma patients. Presence of an effector multigene signature among vaccine responders.	[105]
	High TLS aggregates with increased PD-1/PD-L1 signaling as centers for generation of adaptive antitumor responses upon vaccination with GVAX in patients with PDAC. Implications for combining GVAX with ICIs.	[106]
	Murine glioblastoma regression associated with large sized TLS infiltrated by high numbers of effector-memory T cells and by activated B cells upon vaccination with HCMV-IE1.	[108]
TLS in TME (ICIs-induced)	Reduced melanoma growth upon ICIs therapy via development of TLS infiltrated by T cells, B cells, and other tumor reactive immune elements upon therapy with ICIs.	[99,100,101]

TME, tumor microenvironment; Tfh, follicular helper T cell; TLS, tertiary lymphoid structures; PD-1, programmed cell death protein-1; PD-L1, PD-ligand 1; ICIs, immune checkpoint inhibitors; NSCLC, non-small cell lung cancer; MAGE, melanoma-associated antigen; IDO, indoleamine 2,3-dioxygenase; GVAX, GM-CSF-secreting tumor vaccine; HCMV, human cytomegalovirus; IE1, immediate-early protein 1; GC, gastric cancer; CCA, cholangiocarcinoma; ccRCC, clear cell renal cell carcinoma; RCC, renal cell cancer; PDAC, pancreatic ductal adenocarcinoma; HCC, hepatocellular carcinoma.

## Data Availability

No new data were created or analyzed in this study.

## Abbreviations

The following abbreviations are used in this manuscript:

TLS	tertiary lymphoid structures
TME	tumor microenvironment
DCs	dendritic cells
GCs	germinal centers
HEVs	high endothelial venules
BCa	breast cancer
TNBC	triple-negative breast cancer
TILs	tumor infiltrating lymphocytes
PD-1	programmed cell death protein-1
PD-L1	programmed cell death protein-1 ligand
NSCLC	non-small cell lung cancer
CXCL	chemokine C-X-C motif ligand
CCL	chemokine (C-C motif) ligand
ICIs	immune checkpoint inhibitors
FDCs	follicular dendritic cells
FRCs	fibroblastic reticular cells
Tfh	follicular helper T cell
IL	interleukin
NAC	neoadjuvant chemotherapy
TC	tumor center
IM	invasive margin
OS	overall survival
DFS	disease-free survival
FCIS	favorable combined immune signature
UCIS	unfavorable combined immune signature
RFCIS	reinforced favorable combined immune signature
RUCIS	reinforced unfavorable combined immune signature
CTLA-4	cytotoxic T-lymphocyte-associated protein 4
IDO-1	indoleamine 2,3-dioxygenase 1
CIN	cervical intraepithelial neoplasia
HCMV	human cytomegalovirus
IE1	immediate-early protein 1
PDAC	pancreatic ductal adenocarcinoma
